# Prospective cohort of adult oral health in Piracicaba, SP, Brazil

**DOI:** 10.1186/s13104-019-4243-y

**Published:** 2019-04-11

**Authors:** Manoelito Ferreira Silva-Junior, Maria da Luz Rosário de Sousa, Marília Jesus Batista

**Affiliations:** 1 Department of Dentistry, State University of Ponta Grossa, Avenue General Carlos Cavalcanti, 4748, Ponta Grossa, PR Zip Code: 84.030-900 Brazil; 2 Department of Community Dentistry, Piracicaba Dental School, University of Campinas, Avenue Limeira, 901, P.O. Box: 52, Piracicaba, SP Zip Code: 13414-018 Brazil; 3 Community Health, Jundiaí Medical School, Street Francisco Telles, 250, P.O. Box: 1295, Jundiaí, SP Zip Code: 13202-550 Brazil

**Keywords:** Oral health, Adult, Tooth loss, Health literacy, Epidemiology

## Abstract

**Objective:**

To describe the methodological aspects of a Prospective Cohort Study of adult oral health in Piracicaba, Brazil.

**Results:**

This Prospective Cohort Study evaluated adults (20–64 years old) between the years of 2011 and 2015, in Piracicaba, São Paulo, Brazil. The main objective was to evaluate the risk factors for tooth loss in adults. Data were collected at households and selected via probabilistic sampling, through clinical examination of caries, considering as variables the decayed, missing and filled permanent teeth index, need for caries treatment, periodontal disease (Community Periodontal Index and Periodontal Attachment Loss), use and need for dental prosthesis, and presence of visible biofilm. A questionnaire about demographic, socioeconomic and health habits, use of dental services, self-perceived quality of life (Oral Health Impact Profile-14) and health literacy (14-item Health Literacy Scale) was also employed. In 2011, 248 adults participated, and in 2015, 143 (follow-up rate = 57.7%). Despite the follow-up sample loss, most sociodemographic characteristics remained in the participant sample: for example, women (72.0%) (p = 0.534), family income between R$545,00 and R$1090,00 (63.9%) (p = 0.920), above 11 years of education (53.1%) (p = 0.200) and belonging to middle class (67.1%) (p = 0.909).

## Introduction

According to the Global Burden of Disease, between 1990 and 2010 the conditions of untreated caries, severe periodontal disease, and tooth loss were among the 100 conditions with the most impact on worldwide health [1]. Oral diseases, in addition to causing physical, social and psychological injuries, have a financial impact on developed as well as developing countries [2]. Even though they are preventable, they remain one of the major public health problems in the world [3].

Despite significant advances in the field of Dentistry and Public Health, untreated caries in permanent teeth is the most prevalent disease in the world [4], with periodontal disease in the sixth place [5]. In both cases, the last decades have seen no reduction of prevalence in adults [4, 5], and these health issues commonly lead to tooth loss [1].

Epidemiological surveys are an important instrument for verifying the actual health conditions of the population and the general effects of provided services [6]. Recent studies have shown a reduction in the impact of oral diseases to younger age groups. Their prevalence and severity, however, increases depending on the studied age group [1, 4, 5]. Due to disparities in the experience of caries (DMFT) found in the adult age group (35–44 years) by the last epidemiological surveys of oral health performed in Brazil [6] when compared with adolescents (15–19 years) and elderly (65–74 years), it is necessary to study an extended adult age group and not as recommended by the World Health Organization [7].

Thus, to gather data on the incidence, distribution and risk factors associated with the main oral diseases, it is necessary to conduct a prospective cohort of oral health in an extended adult age group. This can contribute to health promotion actions and reduce impacts on the population’s quality of life [8]. To this date, no longitudinal population-based study of the adult age group in Brazil has been found. This study aims to describe methodological aspects involved in a Prospective Cohort Study of adult oral health in Piracicaba, Brazil. The main objective of this cohort was to evaluate the risk indicators for tooth loss in adults.

## Main text

### Methods

#### Study design

Starting from a 2011 baseline, this Prospective Cohort Study was performed with adults between 20 and 64 years old, in Piracicaba, São Paulo, Brazil.

#### Ethical aspects

This research was approved by the Research Ethics Committee of the Piracicaba Dental School, Campinas State University (Number 177/2009).

#### Population and sample

Sample selection was based on the Brazilian Demographic Census (2000) [9], the latest data available at the time of the study. Piracicaba, SP, Brazil, had 202,131 adults in the 20–64 years age group. The sample size were calculated in two age groups: 20–44 years old and 45–64 years old, because of the difference in oral health conditions between these two groups. Effect design (deff) was 1.5; 10.0% of margin of error, 95% of confidence interval and prevalence of caries experience among the adults (70.2% for young adults and 90.9% for older adults) [10]. The total sample size was 240 adults. It was added 30% to the total to compensate occasional sample loss and effects of non-response, resulted in the need for participation of 342 adults [8, 11].

The sample selection occurred in two stages. First a total of 30 census tracts, number used in Brazilian oral health epidemiological studies, were randomly selected from 392 in this municipality, using probability sampling, plus 2 in case some needed to be replaced. It was included in the study census tracts in urban area and no special characteristics. In the second stage, considering the selection of an adult per household, was considered for the calculation the average number of residents per household was 2.5 and number of households per sector was 177.8 in the 2000 census [9]. Thus, to achieve the number of adults in the sample calculus, an average of 11.4 households per census tract would be required [8, 11]. The households were selected considering the fraction determined by the number of total households divided per 11. An example, if the census presented 220 houses, it was defined the fraction 220 divided per 11, resulting in the fraction of number of houses to selected one, in this case, for each 20 houses travelled, the examiner selected 1 house, varying according the number of houses of census tract [8, 11].

#### Data collection

##### Baseline

Data collection was carried out between June and September 2011. Inclusion criteria were: participants had to be living in selected residences in Piracicaba, São Paulo, and had to be between 20 and 64 years old (in 2011). Exclusion criteria were: unable to participate due to physical and psychological conditions [8, 11]. When the household had been empty at three attempts to contact the participant, it was considered a lost.

During the baseline stage, one examiner conducted the study, after being trained by a standard examiner, during 16 h. Intraexaminer agreement ranged from 96.5 to 100.0%, and the Kappa coefficient ranged from 0.89 to 1.00 [8, 11].

The research consisted of clinical oral examinations and interviews, as shown in Table 1. Clinical examinations took place in the households, under artificial lighting without prior prophylaxis or drying, using CPI-probes and front surface mouth mirrors, as recommended by the World Health Organization [7]. In addition, each participant answered a self-administered questionnaire.

**Table 1:** Epidemiological measures evaluated in the study

Stage	Epidemiological measures
Baseline (2011)	*Questionnaire* Demographic and socioeconomic factors: sex, age, race, self-declared, marital status, individual and family income, number of family members, education, condition of housing, occupation Factors related to general health: health conditions, use of medicines and health habits Factors related to oral health: oral hygiene, self-perception of oral health, reason for dental extraction, access to oral health services, self-perceived quality of life (Oral Health Impact Profile-14) [14] and oral health literacy [15] *Clinical exam* Visible biofilm [16] Experience of coronal caries (DMFT) [7] Need for dental caries treatment [7] Community Periodontal Index (CPI) [7] Periodontal Attachment Loss (PAL) [7] Use and need of dental prosthesis [7]
Folow-up (2015)	*Questionnaire*: same conditions assessed during baseline stage (2011) and addition of questions on the perception of the spouse’s oral health and Health Literacy (14-items Health Literacy Scale) [17, 18] *Clinical exam*: same conditions assessed during baseline stage (2011) and inclusion of evaluation of experience of root caries [7] *Anthropometric measurements*: weight, height and abdominal circumference

##### Follow-up

Data collection took place between June and September 2015. Inclusion criterion was having participated in the baseline independent of actual address. Exclusion criteria were the same as the baseline [12, 13]. Details of calibration process were described in previous publication [13].

Subjects were sought at their addresses and invited to participate in the study. The research consisted of clinical oral examinations and interviews using the same criteria and examination protocol employed during baseline stage [8, 11], as well as new exam, as shown in Table 1. In addition, each volunteer answered a self-administered questionnaire, containing additional research questions. At the time of data collection, each subject kept the same baseline identification [12, 13].

### Results

**Fig. 1:**
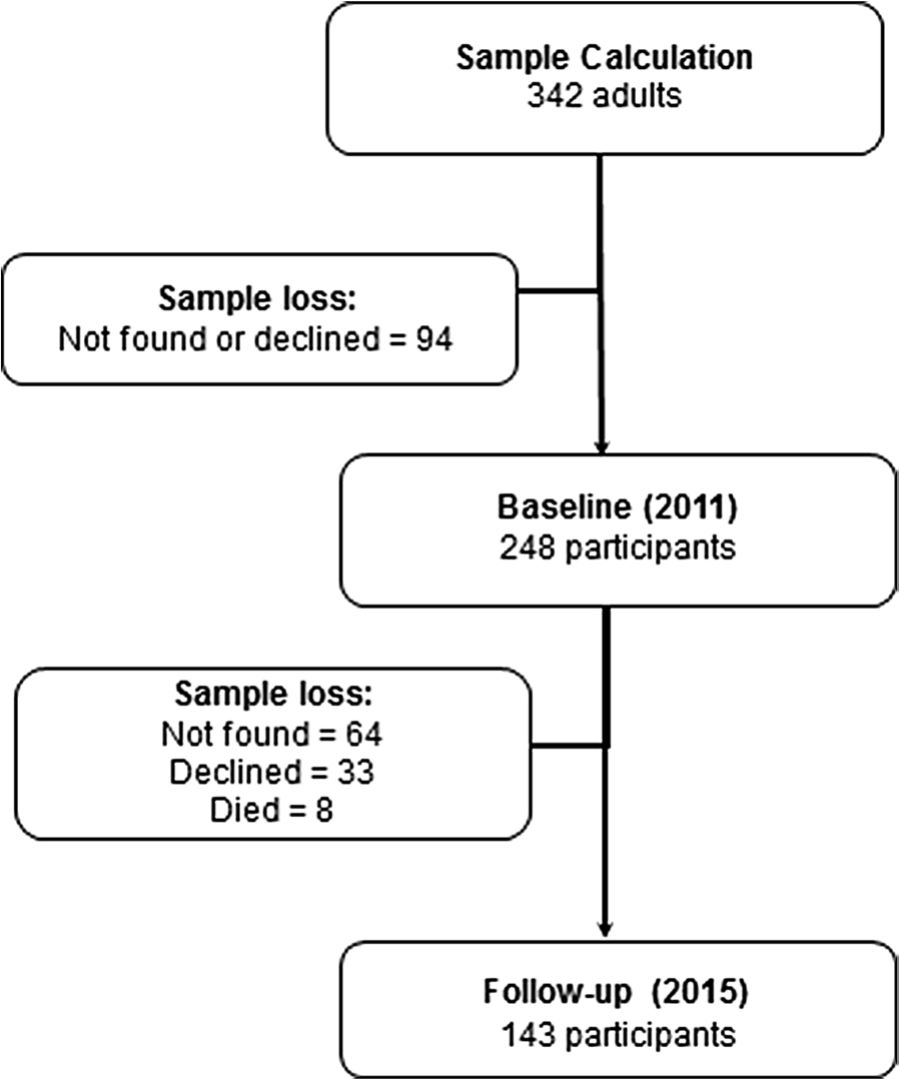
Flowchart of sample size and sample loss (baseline and follow-up, 4 years later). Piracicaba, SP, Brazil, 2011–2015

During baseline stage, there was a loss of 94 (24.0%) adults because they did not agree to participate in the study or were not found (Fig. 1). The baseline sample was composed of 248 adults, representing 149,635 residents adults between 20 and 64 years old from Piracicaba, São Paulo, Brazil.

During follow-up stage, the sample was composed of 143 (follow-up rate = 57.7%) individuals. The reasons for not participating were: 64 (25.8%) could not be found, 33 (13.3%) refused to participate and 8 (3.2%) died (Fig. 1).

Despite the occurrence of sample loss at follow-up (Fig. 1), women (72.2% and 72.0%, respectively), subjects with an income between 1 and 2 minimum wages (R$545,00–R$1090,00) (64.9% and 63.9%), subjects who had above 11 years of education (54.8% and 53.1%), and subjects who belonged to the middle class (67.4% and 67.1%) remained in the participating sample (Table 2).

**Table 2:** Bivariate analysis of demographic and socioeconomic characteristics in the initial and final sample of adults, participants and non-participants, residing in Piracicaba, SP, Brazil, 2011–2015

Characteristics^a^	2011	2015		p-value*****
	Sample	Non-participant	Participant	
	n (%)	n (%)	n (%)	
Demographic				
Sex				
Male	69 (27.8)	29 (27.6)	40 (28.0)	0.534
Female	179 (72.2)	76 (72.4)	103 (72.0)	
Age^b^				
Age range 1	138 (55.6)	68 (64.8)	70 (49.0)	*0.013*
Age range 2	110 (44.4)	37 (35.2)	73 (51.0)	
Race				
White	198 (79.8)	76 (72.4)	122 (85.3)	*0.012*
Not white	50 (20.2)	29 (27.6)	21 (14.7)	
Marital status				
Stable union	174 (70.2)	62 (59.0)	112 (78.3)	*0.001*
No stable union	74 (29.8)	43 (40.1)	31 (21.7)	
Socioeconomic				
Household income^c^				
≤ 1 minimum wage	39 (15.7)	16 (15.2)	23 (16.1)	
1–2 minimum wages	161 (64.9)	70 (66.7)	91 (63.6)	0.920
≥ 2 minimum wages	42 (17.0)	17 (16.2)	25 (17.5)	
Not informed	06 (2.4)	02 (1.9)	04 (2.8)	
Education level				
≤ 4 years	43 (17.4)	13 (12.4)	30 (21.0)	0.200
5–10 years	69 (27.8)	32 (30.5)	37 (25.9)	
≥ 11 years	136 (54.8)	60 (57.1)	76 (53.1)	
Socioeconomic class^d^				
Low	38 (15.3)	15 (14.3)	23 (16.1)	0.909
Middle	167 (67.4)	71 (67.6)	96 (67.1)	
High	43 (17.3)	19 (18.1)	24 (16.8)	

Italic values indicate significance of p value (p < 0.05)

*****Qui-square (p < 0.05)

^a^Considering baseline age (2011)

^b^Age range 1 (20–44 years old) and age range 2 (45–64 years old) in 2011

^c^Minimum wage = R$545,00 (2011)

^d^Socioeconomic classification was performed according to Graciano et al. [19], using a score based on schooling, family income, occupation, type of residence and number of residents in the household, and results in six social classes, in the present study, were grouped into three categories: low, middle and high

### Discussion

This is the first prospective population-based cohort study on oral health in adults in Brazil, and the information from this cohort can be verified in recent publications, whether in respect to baseline (first-wave) [8, 11, 13, 20–24] and second-wave data [12, 13, 18, 25].

Although it had follow-up losses, this study is in line with other longitudinal studies [26–28]. Loss of follow-up was aggravated by the fact that this was a population-based study in the adult age group, with a follow-up period of nothing less than 4 years. When working with a predominantly economically-active age group, there is a methodological difficulty in finding participants in their residences, for data collection, and in reencountering them in the same household, after a long period.

The greater participation of women has been verified among surveys employing household data collection [6, 29]. This is not what happens in studies with workers, in which there is a greater participation of men [10]. This raises questions on the best place for data collection in the adult age group: one must consider the representativeness of the population and at the same time minimize possible biases of sample selection [22].

During follow-up, although socioeconomic characteristics were the same in the participant and non-participant sample, there were statistical differences in the prevalence of some of these characteristics. Older individuals with white skin color and stable union predominated in the final sample. One possible explanation is that individuals with greater financial stability, such as house owners, have a greater chance of remaining in the sample.

This cohort study’s initial research line was concerned with data related to dental loss, such as: a new classification of tooth loss [20], incidence description [12, 13] and risk factors [12], impact on self-perceived quality of life [8, 11], self-reported reasons for tooth extraction [12, 22], and spatial distribution [23, 24]. Currently, studies have been focusing on the association between health literacy and health behaviors, self-perceived quality of life and oral clinical conditions [21, 25]. For this, the cross-cultural adaptation and validation of the *14*-*item Health Literacy Scale* (HLS-14) can be a useful instrument, capable of evaluating health literacy in three levels: functional, communicative and critical [18].

## Conclusion

It was described the methodological aspects of the oral health cohort of adults, that was very important to better understand the risk of tooth loss and others aspects involved with oral health.

## Limitations

- During baseline stage, household collection led the sample to be composed mostly of women.
- During follow-up stage, there was sample loss, and greater participation of older individuals, with white skin color and in stable union.
- During the follow-up stage, the gross age was not considered but which age group the individual belonged at baseline in order to keep the adult in the study and did not change it from their subgroup.
- In this longitudinal study, a new sample calculation was not performed, because the objective was to evaluate the variables in the same individuals from the initial sample. This may not refer to the representativeness of the initial population.
- In two stages, the data collection was performed by interview and not by self-administered questionnaire, since there were illiterate individuals who were not excluded from the sample. Thus, the standardization by this means of collection by interview was adequate to the participation of all the universe chosen for the study.
- In two stages, the presence of periodontal pocket, it should be noted that this measure was performed by index teeth in each sextant, and not individualized.

Despite the limitations presented in the study, the data are reliable to present results referring to a population epidemiological study of oral health of adults, and it is encouraged that future studies may overcome the methodological limitations presented here.

## Abbreviations

CPI: Community Periodontal Index
DMFT: Decayed, Missing, Filled Teeth Index
PAL: Periodontal Attachment Loss
WHO: World Health Organization

## References

[1] Kassebaum NJ, Bernabé E, Dahiya M, Bhandari B, Murray CJ, Marcenes W (2014). Global burden of severe periodontitis in 1990–2010: a systematic review and meta-regression. J Dent Res.

[2] McGrath C, Lawrence HP, Blinkhorn A (2012). Guest editorial on the Festchrift “challenges in population oral health for the 21st century”. Community Dent Oral Epidemiol.

[3] Petersen PE, Kwan S, Blinkhorn A (2012). The 7th WHO Global Conference on Health Promotion—towards integration of oral health. Community Dent Health.

[4] Kassebaum NJ, Bernabé E, Dahiya M, Bhandari B, Murray CJ, Marcenes W (2015). Global burden of untreated caries: a systematic review and metaregression. J Dent Res.

[5] Kassebaum NJ, Bernabé E, Dahiya M, Bhandari B, Murray CJL, Marcenes W (2014). Global burden of severe tooth loss: a systematic review and meta-analysis. J Dent Res.

[6] Nascimento S, Frazão P, Bousquat A, Antunes JLF (2013). Condições dentárias entre adultos brasileiros de 1986 a 2010. Rev Saude Publica.

[7] World Health Organization (1997). Oral heath surveys: basic methods.

[8] Batista MJ, Lawrence HP, Sousa MLR (2014). Impact of tooth loss related to number and position on oral health quality of life among adults. Health Qual Life Outcomes.

[9] Instituto Brasileiro de Geografia e Estatísticas (IBGE). Censo Demográfico 2000. Brasília: IBGE; 2000. Disponível em: http://www.ibge.gov.br/censo. Acesso em 1 Mar 2010.

[10] Batista MJ, Rihs LB, Sousa MLR (2012). Risk indicators for tooth loss in adult workers. Braz Oral Res..

[11] Batista MJ. Impacto da perda dentária na qualidade de vida de adultos. Piracicaba-SP. 2013. Tese [Doutorado em Odontologia]. Piracicaba: Faculdade de Odontologia de Piracicaba da Universidade Estadual de Campinas; 2013.

[12] Silva-Junior MF. Estudo longitudinal das perdas dentárias em adultos e fatores associados. 2016. Dissertação [Mestrado em Odontologia]. Piracicaba: Faculdade de Odontologia de Piracicaba da Universidade Estadual de Campinas; 2016.

[13] Silva-Junior MF, Batista MJ, Sousa MLR (2017). Incidence of tooth loss in adults: a population-based prospective cohort study. Int J Dent..

[14] Oliveira BH, Nadanovsky P (2005). Psychometric properties of the Brazilian version of the Oral Health Impact Profile-short form. Community Dent Oral Epidemiol.

[15] Ishikawa H, Nomura K, Sato M, Yano E (2008). Developing a measure of communicative and critical health literacy: a pilot study of Japanese office workers. Health Promot Int.

[16] Ainamo J, Bay I (1975). Problems and proposals for recording gingivitis and plaque. Int Dent J.

[17] Suka M, Odajima T, Kasai M, Igarashi A, Ishikawa H, Kusama M, Nakayama T, Sumitani M, Sugimori H. The 14-item health literacy scale for Japanese adults. 10.1007/s12199-013-0340-z PMC3773092 23689952

[18] Marques ACP. Tradução, adaptação transcultural e validação de um instrumento de literacia em saúde para adultos. Piracicaba-SP. Dissertação [Mestrado Profissional em Odontologia em Saúde Coletiva]. Universidade Estadual de Campinas, Faculdade de Odontologia de Piracicaba; 2016.

[19] Graciano MIG, Lehfeld NA, Neves Filho A (1999). Critérios de avaliação para classificação socioeconômica: elementos de atualização. Serviço Social & Realidade..

[20] Batista MJ, Lawrence HP, Sousa MLR (2015). Classificação das perdas dentárias: fatores associados a uma nova medida em uma população de adultos. Ciênc Saúde Coletiva..

[21] Batista MJ, Lawrence HP, Sousa MLR (2018). Oral health literacy and oral health outcomes in an adult population in Brazil. BMC Public Health..

[22] Silva-Junior MF, Souza AAC, Batista MJ, Sousa MLR (2017). Condição de saúde bucal e motivos para extração dentária entre uma população de adultos (20-64 anos). Ciênc Saúde Coletiva..

[23] Silva-Junior MF, Batista MJ, Fonseca EP, Sousa MLR (2017). Spatial distribution of tooth loss in a population of adults. Rev Gaúcha Odontol..

[24] Silva-Junior MF, Batista MJ, Fonseca EP, Sousa MLR (2019). Spatial distribution of tooth loss in a population of adults. Rev Gaúcha Odontol..

[25] Silva-Junior MF. Impacto da literacia em saúde nos comportamentos e condições clínicas de saúde bucal em uma coorte de adultos e idosos: um estudo quanti-qualitativo. 2018. Tese [Doutorado em Odontologia]. Piracicaba: Faculdade de Odontologia de Piracicaba da Universidade Estadual de Campinas; 2018.

[26] Haas AN, Gaio EJ, Oppermann RV, Rösing CK, Albandar JM, Susin C (2012). Pattern and rate of progression of periodontal attachment loss in an urban population of South Brazil: a 5-years population-based prospective study. J Clin Periodontol.

[27] Gustavson K, von Soest T, Karevold E, Røysamb E (2012). Attrition and generalizability in longitudinal studies: findings from a 15-year population-based study and a Monte Carlo simulation study. BMC Public Health..

[28] van der Velden U, Amaliya A, Loos BG, Timmerman MF, van der Weijden FA, Winkel EG, Abbas F (2015). Java project on periodontal diseases: causes of tooth loss in a cohort of untreated individuals. J Clin Periodontol.

[29] Pinto RS, Matos DL, Filho AIL (2012). Características associadas ao uso de serviços odontológicos públicos pela população adulta brasileira. Cien Saude Coletiva.

