# Correction to: Prospective cohort of adult oral health in Piracicaba, SP, Brazil

**DOI:** 10.1186/s13104-019-4522-7

**Published:** 2019-08-14

**Authors:** Manoelito Ferreira Silva-Junior, Maria da Luz Rosário de Sousa, Marília Jesus Batista

**Affiliations:** 1 Department of Dentistry, State University of Ponta Grossa, Avenue General Carlos Cavalcanti, 4748, Ponta Grossa, PR Zip Code: 84.030-900 Brazil; 2 Department of Community Dentistry, Piracicaba Dental School, University of Campinas, Avenue Limeira, 901, P.O. Box: 52, Piracicaba, SP Zip Code: 13414-018 Brazil; 3 Community Health, Jundiaí Medical School, Street Francisco Telles, 250, P.O. Box: 1295, Jundiaí, SP Zip Code: 13202-550 Brazil

## Correction to: BMC Res Notes (2019) 12: 221 10.1186/s13104-019-4243-y

The original publication of this article [1] did not include the funding acknowledgement of FAPESP. The authors would like to acknowledge the funding by **FAPESP**.

In addition, references 18 and 24 did not contain all correct bibliographic information. The updated references 18 and 24 are shown below:

18. Batista MJ, Marques ACP, Silva Junior MF, Alencar GP, Sousa MLR. Tradução, adaptação transcultural e avaliação psicométrica da versão em português (brasileiro) do 14-item Health Literacy Scale. Cien Saude Colet. In press. Disponible from: http://www.cienciaesaudecoletiva.com.br/artigos/traducao-adaptacao-transcultural-e-avaliacao-psicometrica-da-versao-em-portugues-brasileiro-do-14item-health-literacy-scale/17070?id=17070
24. Silva-Junior MF, Batista MJ, Fonseca EP, Sousa MLR. Spatial distribution of decayed and restored teeth in an adult population. Rev Gaúcha Odontol. 2019;67:e2019006.
